# Enterocolic Lymphocytic Phlebitis Treated Preoperatively with Biologics and Immunosuppressive Agents

**DOI:** 10.1155/2022/5120607

**Published:** 2022-03-07

**Authors:** Soh Okano, Takashi Yao, Osamu Nomura, Akihito Nagahara, Toshiaki Hagiwara, Kiichi Sugimoto, Makoto Takahashi, Kazuhiro Sakamoto

**Affiliations:** ^1^Department of Human Pathology, Graduate School of Medicine, Juntendo University, 2-1-1 Hongo, Bunkyo-Ku, Tokyo 113-8421, Japan; ^2^Department of Gastroenterology, Graduate School of Medicine, Juntendo University, 2-1-1 Hongo, Bunkyo-Ku, Tokyo 113-8421, Japan; ^3^Department of Coloproctological Surgery, Graduate School of Medicine, Juntendo University, 2-1-1 Hongo, Bunkyo-Ku, Tokyo 113-8421, Japan

## Abstract

Enterocolic lymphocytic phlebitis is phlebitis of unknown etiology in which lymphocytes affect veins without arteries and shows evidence of systemic vasculitis in the intestinal wall and mesentery, mainly in the small intestine and colon. Although patients present with a variety of gastrointestinal symptoms and findings like those of inflammatory bowel disease or ischemic bowel disease, there are no specific findings for enterocolic lymphocytic phlebitis. As a result, a diagnosis tends to be made after surgery. There are few case reports of enterocolic lymphocytic phlebitis, and the impact of chronic courses and immunosuppressive drugs on enterocolic lymphocytic phlebitis is not well known. A 47-year-old man was treated with infliximab and steroids for unexplained ulceration and narrowing of the ileocecal area, which was suspected to be inflammatory bowel disease with atypical findings. Lymphocytic phlebitis was noted in the surgical specimen, and enterocolic lymphocytic phlebitis was diagnosed. No recurrence of enterocolic lymphocytic phlebitis was observed postoperatively. This disease should also be considered among patients with inflammatory bowel disease-like lesions that do not respond to infliximab or steroids.

## 1. Introduction

Enterocolic lymphocytic phlebitis (ELP) is a lymphocytic phlebitis of unknown etiology that occurs in the mesentery and intestinal wall with predominant infiltration of T cells [1]. It does not present with systemic phlebitis and does not affect arteries.

Cases of what appear to be the same disease have been reported as mesenteric inflammatory veno-occlusive disease (MIVOD), intramural mesenteric venulitis, lymphocytic venulitis [1], and chronic intestinal lymphocytic microphlebitis [2]. Histologically, cases of granulomatous phlebitis [3], necrotizing phlebitis [4], and myointimal hyperplasia [5] have also been reported.

Gastrointestinal symptoms such as abdominal pain, nausea and vomiting, diarrhea, and gastrointestinal bleeding have been reported in some cases [5–13]. The small and large intestines are the most common sites of diagnosis [1, 9, 14], and the disease may resemble ischemic or inflammatory bowel disease (IBD) [6, 7]. However, due to the lack of disease-specific endoscopic or imaging findings [1], the diagnosis is made postoperatively, not preoperatively.

There is no established method for preoperative diagnosis of ELP. In addition, medical treatments other than surgery are unknown.

In this study, we encountered a case of ELP that was not diagnosed preoperatively but had been treated medically as Crohn's disease due to its long-term course. It is rare to find a case with a long-term history of ELP that underwent medical treatment prior to surgery, and the histology of this case is meaningful when considering the effect of the treatment.

## 2. Case Presentation

A 47-year-old man had been experiencing abdominal pain for a year. The patient underwent colonoscopy (CS) and computed tomography (CT) performed by a local doctor, which revealed a huge ulcer in the ileocecal region and associated narrowing of the ileocecal region. Biopsy and culture tests were repeated. However, this did not lead to a definitive diagnosis. Gastrointestinal Behçet's disease or Crohn's disease was suspected.

The patient was treated with infliximab (IFX), 5-aminosalicylic acid (5ASA), colchicine, and budesonide, but the therapeutic agents were not effective enough to achieve remission.

The patient visited our hospital 7 months after the onset of symptoms. At our hospital, double-balloon endoscopy (DBE) and small bowel follow-through (SBFT) were performed, and no foci other than the main lesion were found. First, IFX, 5ASA, and budesonide were continued, but due to lack of therapeutic effect, IFX and budesonide were discontinued five months after presenting to our hospital, and the treatment was switched to ustekinumab (UST). The patient was admitted to the hospital with diarrhea and vomiting, and contrast-enhanced (CE) CT revealed thickening of the wall of the ileum. CECT showed thickening of the ileocecal wall, and culture, T-spot, and C7HRP tests were all negative.

**Figure 1:**
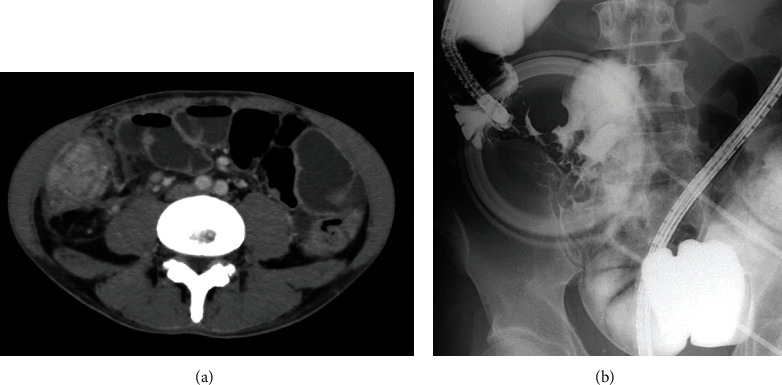
Preoperative radiographic findings. (a) Contrast-enhanced computed tomography: wall thickening with contrast effect is seen in the ileocecal region. (b) Endoscopic retrograde ileography: severe stenosis and deformation of the ileocecal region.

Clinical symptoms were ameliorated with intravenous fluids and fasting, but stenosis of the ileocecal lesion was worse on DBE and SBFT (Figure 1). Endoscopic balloon dilation was performed with little improvement; therefore, the patient underwent ileocecal resection.

**Figure 2:**
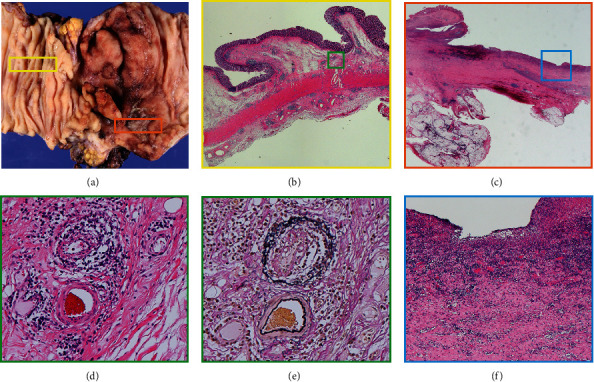
Pathological findings. (a) Gross appearance of resected specimens. Severe stenosis and ulceration are present at the ileocecal region. (b) Low-magnification image of the area surrounded by the yellow frame. Phlebitis is found in the submucosa beneath the normal mucosa away from the main lesion (hematoxylin/eosin (HE) stain). (c) Low-magnification image of the area surrounded by the orange frame (HE stain). (d) Magnified image of the area surrounded by the green frame. Lymphocytic phlebitis is present but the arteries are not infiltrated (HE stain). (e) Elastica van Gieson stain. (f) Magnified image of the area surrounded by the blue frame. In the middle part of the lesion, there was no phlebitis. There were only ulcers and fibrosis (HE stain).

Surgical specimens showed lymphocytic phlebitis in small- to medium-sized veins without arterial infiltration (Figures 2(a), 2(b), 2(d), and 2(e)). Moreover, there were no other specific findings. Therefore, a diagnosis of ELP was made.

**Figure 3:**
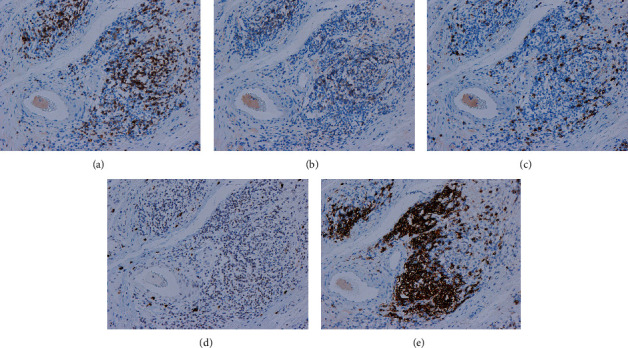
Pathological findings. Immunostaining shows that CD3+, CD4+, and CD8+ T cells mainly infiltrate the veins, and CD20+ B cells infiltrate the surrounding areas. Activated natural killer cells and cytotoxic T cells that are positive for granzyme B have a nonspecific distribution. (a) CD3. (b) CD4. (c) CD8. (d) Granzyme B. (e) CD20.

In addition, the distribution of lymphocytic phlebitis was characteristic. Lymphocytic phlebitis was found in the ascending colon and terminal ileum regions, where the mucosa covering the central and lateral portions of the lesion was normal. In contrast, in the central part of the lesion, only ulcers with severe inflammation and fibrosis were found, and no lymphocytic phlebitis was found (Figures 2(a), 2(c), and 2(f)). Histologically, lymphocytic phlebitis consisted of CD3+/CD8+ T cells infiltrating the vein wall and CD20+ B cell lymphocytes in the periphery of the vein (Figure 3).

The patient had a good postoperative course and was discharged from the hospital. Since then, no recurrence of ELP has been observed.

## 3. Discussion

Two characteristics of ELP are shown in this case. First, regarding the diagnosis, the veins in the intestinal wall in the area slightly outside the main locus of the lesion need to be observed to determine findings of ELP. Second, immunosuppressive drugs may not be effective in treating ELP.

It would be ideal to diagnose ELP preoperatively. However, ELP has no specific findings from endoscopy or imaging studies [1]. The only specific finding, lymphocytic phlebitis, is unlikely to be collected by biopsy. This makes diagnosis difficult [14].

ELP is often associated with an underlying disease [1, 5, 7, 15, 16]. Therefore, in cases of undiagnosed IBD-like lesions with underlying disease, it is important to consider treatment options that also include ELP. In other words, when patients present with intractable disease conditions such as stricture, as in this case, bowel resection should be considered.

In fact, many cases of ELP are diagnosed after bowel resection [5–8, 10–12, 17, 18] because the entire wall of the intestine can be assessed by bowel resection to detect venous-specific lymphocytic phlebitis.

In this case, the histological findings of ELP were concentrated in the outer normal submucosal area of the lesion, and the central area was mainly characterized with ulceration and fibrosis with no phlebitis findings. Because of the relatively long history of this case from the onset of the disease to the surgery, it is likely that any specific findings had disappeared due to chronic secondary changes in the central region.

Therefore, to diagnose ELP, it may be helpful to look at the veins in the intestinal wall in the area slightly outside the main site of the lesion.

Secondly, there is no established medical treatment for ELP. The cause of ELP is unknown, but it has been reported to be associated with diversion colitis in the context of IBD [19]. Diversion colitis is a condition caused by a surgical diversion of the flow of feces from the colorectal mucosa. And it also suggests that the intestinal flora may be altered [20].

Therefore, it is expected that an autoimmune mechanism may be associated with changes in the intestinal microbiota [19]. In addition, there are reports of the onset of ulcerative colitis in the background [8] and cases of ulcerative colitis after surgery for ELP, which may be related to ulcerative colitis [11]. These results suggest that drugs for IBD may influence ELP.

However, immunosuppressive drugs such as IFX, UST, and steroids were used preoperatively in this case with no clinical improvement in the condition, and the specific findings from the surgical specimens indicated that these drugs may not be effective for ELP.

In conclusion, for lesions that do not respond to IFX or UST and show a tendency toward narrowing, it is important to consider surgery for definitive diagnosis.

Furthermore, recurrence after surgery for ELP is very rare [1, 14]; hence, surgery may free the patient from symptoms and ineffective treatment.

There have been few reports on ELP. Moreover, the cause of ELP is unknown, and diagnostic and therapeutic methods have not yet been fully established. Therefore, a further accumulation of cases is necessary.
